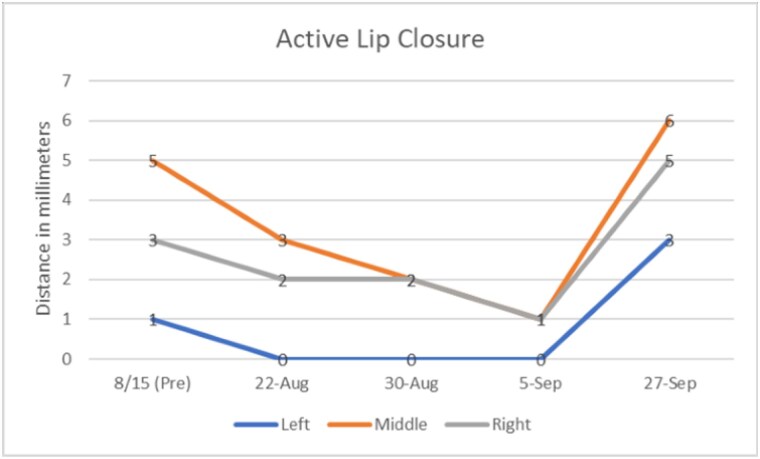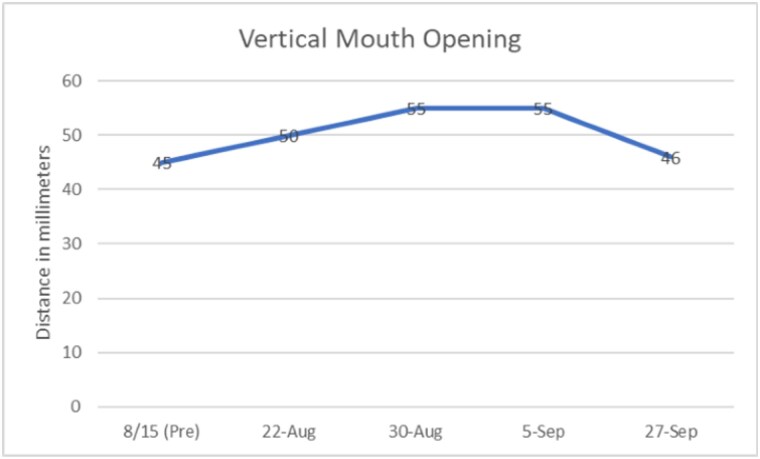# 683 Custom Intra Oral Orthosis: A Novel Device to Treat Lip Ectropion Related to Burn Scar

**DOI:** 10.1093/jbcr/iraf019.312

**Published:** 2025-04-01

**Authors:** Charles Mckenzie, Renee Warthman, Whitney Pirsig, Derek Murray, Karen Richey, Kevin Foster, Arpana Jain

**Affiliations:** Valleywise Health; Diane & Bruce Halle Arizona Burn Center; Diane & Bruce Halle Arizona Burn Center; Diane & Bruce Halle Arizona Burn Center; Diane & Bruce Halle Arizona Burn Center; Diane & Bruce Halle Arizona Burn Center; Diane & Bruce Halle Arizona Burn Center

## Abstract

**Introduction:**

Burn scars around the lip present difficult challenges including mouth opening, closing, management of oral secretions, cosmesis, eating, and nutrition maintenance. Management is frequently confounded due to inability to keep orthosis in proper position. Traditional microstomia prevention appliances (MPAs) require removal for daily activities (eating, drinking, brushing teeth, talking) and wearing schedule is generally shorter applications several times a day. This is a case report of a custom tooth-based intraoral orthosis to effect cutaneous stretching.

**Methods:**

A 45-year-old male suffered 34% TBSA flame burns to face, both upper extremities and chest, resulting in bilateral upper extremity amputations during his acute care phase as well as evisceration of right eye. He also developed severe perioral scar contracture, and lower lip ectropion which interfered with eating, oral secretion management, and speech. He was reliant on assistance from caretakers to place orthotics on face and/or limbs. He underwent a scar release with full thickness skin graft to lower lip ectropion, and subsequent application of a custom intra oral orthosis. Dental impressions were taken for fabrication of the appliance. The design was communicated to the lab with the use of stone models. A metal framework was designed to support the acrylic that will support the labial tissues.

**Results:**

The patient was able to safely wear the appliance the entire day because the appliance was tooth supported, like a traditional partial denture. Lip closure and vertical mouth opening measurements were obtained and the Mouth Impairment Disability Assessment were administered prior to device implementation. Measurements were also noted at the weekly intervals. The patient gained 4 mm of active lip closure and 11 mm in mouth opening over a period of 3 weeks. He gained 2mm on the right side with lip closure and full closure on the left side during the same time. There was also improvement noted in speech and management of oral secretions. At the recent follow up, the patient reported noncompliance with the orthosis for a week. This was reflected in the measurements as loss of previous gains in mouth closure.

**Conclusions:**

A custom intra-oral tooth supported device facilitated improvement in mouth closure, and was easier for the patient to manage, resulting in more therapeutic effect.

**Applicability of Research to Practice:**

This novel custom tooth-based device for intra oral use uncovers a new approach to treat the intractable problem of lower lip burn scar ectropion that is easier to apply and can be worn for longer durations. This is especially important in the presence of other mobility challenges as frequently seen in large body surface are burn injuries.

**Funding for the Study:**

N/A